# Real-time ultrasound elastography in 180 axillary lymph nodes: elasticity distribution in healthy lymph nodes and prediction of breast cancer metastases

**DOI:** 10.1186/1471-2342-12-35

**Published:** 2012-12-19

**Authors:** Sebastian Wojcinski, Jennifer Dupont, Werner Schmidt, Michael Cassel, Peter Hillemanns

**Affiliations:** 1 Hannover Medical School, Department for Obstetrics and Gynecology, OE 6410, Carl-Neuberg-Straße 1, Hannover 30625, Germany; 2 Main-Taunus-Kreis Hospital, Department for Obstetrics and Gynecology, Bad Soden, Germany; 3 University Hospital of Saarland, Department for Obstetrics and Gynecology, Homburg/Saar, Germany; 4 University of Potsdam, Center for Sports Medicine, Recreational and High Performance Sports, Potsdam, Germany

**Keywords:** Breast ultrasound, Axillary lymph nodes, Sonoelastography, Real-time tissue elastography, Cancer detection, Elasticity imaging, HI-RTE, Lymph node metastases

## Abstract

**Background:**

To determine the general appearance of normal axillary lymph nodes (LNs) in real-time tissue sonoelastography and to explore the method′s potential value in the prediction of LN metastases.

**Methods:**

Axillary LNs in healthy probands (n=165) and metastatic LNs in breast cancer patients (n=15) were examined with palpation, B-mode ultrasound, Doppler and sonoelastography (assessment of the elasticity of the cortex and the medulla). The elasticity distributions were compared and sensitivity (SE) and specificity (SP) were calculated. In an exploratory analysis, positive and negative predictive values (PPV, NPV) were calculated based upon the estimated prevalence of LN metastases in different risk groups.

**Results:**

In the elastogram, the LN cortex was significantly harder than the medulla in both healthy (p=0.004) and metastatic LNs (p=0.005). Comparing healthy and metastatic LNs, there was no difference in the elasticity distribution of the medulla (p=0.281), but we found a significantly harder cortex in metastatic LNs (p=0.006). The SE of clinical examination, B-mode ultrasound, Doppler ultrasound and sonoelastography was revealed to be 13.3%, 40.0%, 14.3% and 60.0%, respectively, and SP was 88.4%, 96.8%, 95.6% and 79.6%, respectively. The highest SE was achieved by the disjunctive combination of B-mode and elastographic features (cortex >3mm in B-mode or blue cortex in the elastogram, SE=73.3%). The highest SP was achieved by the conjunctive combination of B-mode ultrasound and elastography (cortex >3mm in B-mode and blue cortex in the elastogram, SP=99.3%).

**Conclusions:**

Sonoelastography is a feasible method to visualize the elasticity distribution of LNs. Moreover, sonoelastography is capable of detecting elasticity differences between the cortex and medulla, and between metastatic and healthy LNs. Therefore, sonoelastography yields additional information about axillary LN status and can improve the PPV, although this method is still experimental.

## Background

The prediction of axillary lymph node (LN) status remains an important issue in the preoperative assessment of breast cancer patients. Sentinel node biopsy (SNB) is the standard option for women that are staged with a negative nodal status [1-5]. Nevertheless, if axillary metastases are suspected, the success of SNB may be impaired. These patients should still receive axillary LN dissection (ALND) [6,7]. The procedure of radical ALND implies a significant increase in morbidity, such as lymphedema or paresthesia of the arm [8]. Provided that the preoperative assessment was correct, the precision of histological staging by SNB is very high and postoperative morbidity is significantly minimized [9]. Recently, omission of radical ALND in certain cases of positive sentinel nodes has been discussed [10,11].

However, the diagnostic precision of the preoperative assessment of the axillary LN status is far from perfect. Palpation of the axilla lacks sensitivity (SE) as only vast metastases are clinically apparent. Mammography does not fully cover the axillary region and the prediction of the malignant or benign character of LNs is not possible. On the other hand, B-mode ultrasound is known to be a precise method for the examination of the axilla with a SE of 45-73% and a specificity (SP) of 44-100%, depending on the distinct B-mode criteria that are investigated [12,13]. Other imaging methods such as computer tomography (CT), magnetic resonance imaging (MRI), scintimammography and positron emission tomography (PET) have been investigated, but they have all demonstrated no relevant clinical advantage in the evaluation of the axilla. Additionally, they are overly expensive and labor-intensive [14-19].

Therefore, ultrasound remains the most suitable imaging method to assess axillary LNs, although the diagnostic accuracy is still unsatisfactory [20]. Technical advances like sonoelastography, tissue harmonic imaging and increasing frequencies may allow a better differentiation of benign and malignant masses [21-23]. Concerning the evaluation of breast lesions, sonoelastography has demonstrated an improved diagnostic performance when combining this method with B-mode ultrasound [22,24-26]. Sonoelastography has also been performed on cervical [27-29], mediastinal [30,31], celiac or mesenteric [32,33] and inguinal [34] LNs.

However, to the best of our knowledge, no data concerning sonoelastography of axillary LNs were published prior to the studies of Choi et al. (n=64) and Taylor et al. (n=50) in 2011 [35,36]. Therefore, our current results from 165 healthy and 15 metastatic LNs may expand the knowledge in this field of research to a certain degree.

Our primary study objective was to determine the typical color distributions of healthy LNs in the elastogram.

The secondary study objective was an exploratory analysis of the method′s potential value in the prediction of LN metastases when used as an adjunct to conventional B-mode ultrasound.

## Materials and methods

Our study was carried out at the Breast Cancer Center in the University Hospital of Saarland, Homburg/Saar, Germany. The responsible ethics committee did not require additional approval for this non-interventional study design. The study cohort (n=180 LNs) was recruited from patients who attended the outpatient service of our institution.

Healthy patients with no suspicious findings in the breast examination were eligible for the control group (group 1, n=165 LNs). In these patients, we performed the experimental sonoelastography of a randomly chosen axillary LN. Patients with a history of breast surgery concerning a larger resection volume, inflammatory conditions of the breast or systemic infections and skin disorders were excluded.

Patients with histologically-proven breast cancer before treatment were potentially eligible for group 2. In these patients, we performed experimental sonoelastography of an ipsilateral axillary LN. These breast cancer patients (n=33) were scheduled to undergo surgery of the breast and the axilla. Concerning the previously studied LN, we used a skin marker for identification and correlated the pathological size with the ultrasonographic size in order to ensure that this was a representative specimen. Eighteen patients had benign axillary LNs on histological examination. These patients were excluded from analysis. The remaining fifteen patients showed metastases in the previously examined LN. These patients were assigned to the metastatic group (group 2, n=15 LNs).

### Ultrasound examinations and image analysis

The routine examinations were performed by the author SW, a DEGUM (Deutsche Gesellschaft für Ultraschall in der Medizin, German society for ultrasound in medicine) level I certified senior physician in gynecology with four years experience in breast ultrasound [37]. The elastograms were obtained by the author JD, a doctoral fellow at our institution. All examinations were performed with the Hitachi EUB-8500 ultrasound system (Hitachi Medical Systems GmbH, Wiesbaden, Germany) using the Hitachi EUP-L54M probe (50 mm, 6–13 MHz) and the integrated elastography module [38].

First, each LN was measured in two planes (i.e. three axes). Furthermore, we determined the dimension of the cortex and the medulla and performed color Doppler ultrasound. Pathological vascularization was defined as the presence of neoangiogenesis disrupting the capsule of the LN or an increased vascularization of the cortex. Next, experimental sonoelastography was carried out. The region of interest for the elastogram was chosen to encompass a maximum of 30% LN tissue and a minimum of 70% encircling tissue.

Image analysis was conducted by JD. As the analysis was performed before surgery, JD had no information about the final histological diagnosis in group 2. The B-mode and Doppler images of each LN were described by standard methods [39]. Concerning the elastogram, the elasticity distribution of the cortex and medulla were described as the predominant color of the particular anatomical region (red, yellow, green, turquoise or blue).

### Sonoelastography

**Figure 1:**
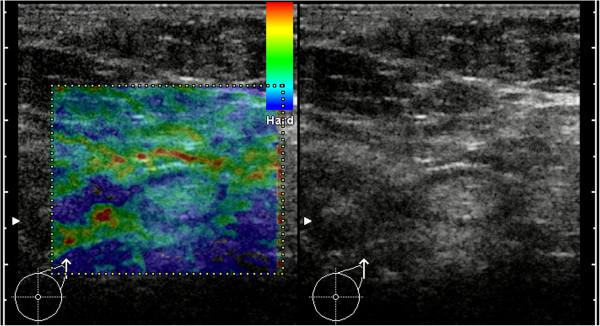
**Example for B-mode ultrasound and elastogram of a healthy LN.** In B-mode ultrasound the LN exhibits no criteria for malignancy. The predominant color of the medulla is green (with smaller areas of turquoise) and the cortex is mainly blue. Applying the criterion of a blue cortex, this case would be a false-positive.

**Figure 2:**
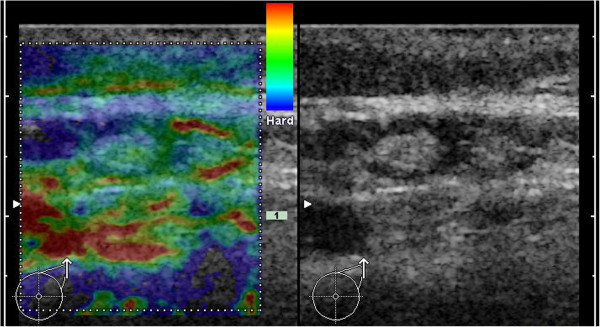
**Example for B-mode ultrasound and elastogram of a healthy LN.** In B-mode ultrasound the LN exhibits no criteria for malignancy. The predominant color of the medulla is turquoise (with smaller areas of green) and the cortex is mainly green. Applying the criterion of a blue cortex, this case would be a true-negative.

**Figure 3:**
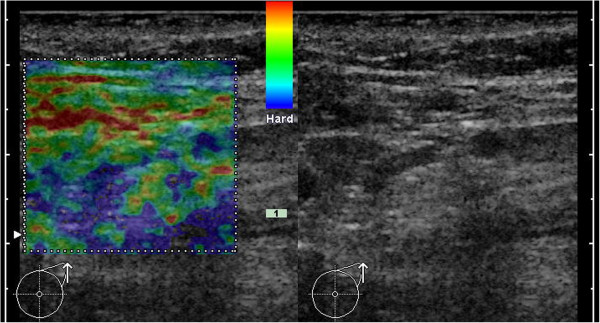
**Example for B-mode ultrasound and elastogram of a healthy, reactive LN.** In B-mode ultrasound the cortex of the LN is slightly enlarge (maximum ~2.5mm). The predominant color of the medulla is green (with smaller areas in other shades) and the cortex is mainly blue (with smaller areas of green). Applying the criterion of a blue cortex, this case would be a false-positive.

**Figure 4:**
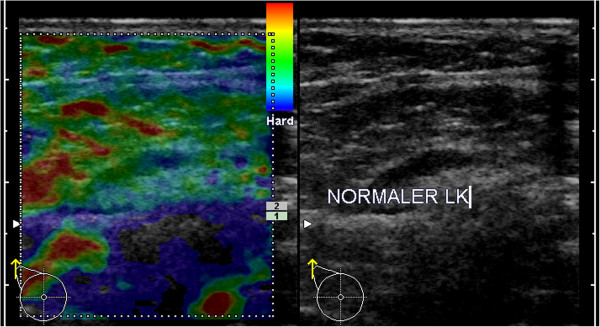
**Example for B-mode ultrasound and elastogram of a healthy, reactive LN.** In B-mode ultrasound, the cortex of the LN is slightly enlarged (maximum ~3.5mm). The predominant color of the medulla is turquoise (to green) and the cortex is mainly green. Applying the criterion of a blue cortex, this case would be a true-negative.

**Figure 5:**
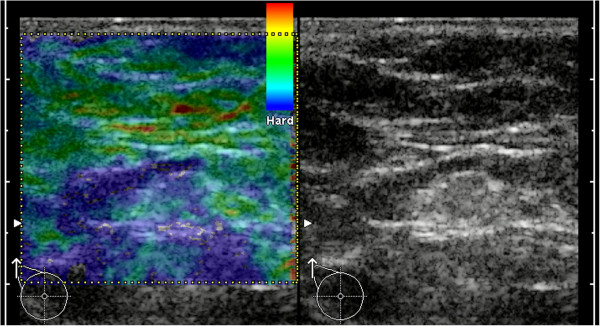
**Example for B-mode ultrasound and elastogram of a metastatic LN.** In B-mode ultrasound, the cortex of the LN is slightly enlarged (maximum ~3.5mm). The predominant color of the medulla is turquoise (to green) and the cortex is mainly blue. Applying the criterion of a blue cortex, this case would be a true-positive.

**Figure 6:**
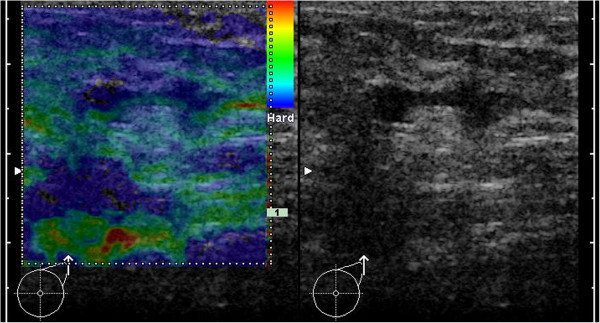
**Example for B-mode ultrasound and elastogram of a metastatic LN.** In B-mode ultrasound, the cortex of the LN is slightly enlarged (maximum ~3.0mm). The predominant color of the medulla is turquoise and cortex is mainly blue. Applying the criterion of a blue cortex, this case would be a true-positive.

Dynamic real-time examinations using ultrasound to access the compressibility of breast lesions were introduced in the 1980s [40]. Today, numerous ultrasound manufacturers offer solutions that include elastography modules in the various ultrasound platforms. The principle of sonoelastography is that the tissue is subjected to a stress (i.e. compression) and the resulting strain (i.e. displacement) is assessed. Typically, the stress is applied by compressing the tissue with the ultrasound probe (freehand/handheld elastography). In addition, the newly developed method of shear wave elastography is under clinical evaluation [41]. This method utilizes an acoustic push pulse (vertically directed) to induce an elastic shear wave (horizontally directed) that propagates through the tissue. The velocity of the shear wave is measured by detection pulses and provides a semi-quantitative measurement of tissue stiffness [42]. In our study, we applied handheld sonoelastography (Hitachi real-time tissue elastography, HI-RTE). This technology provides color elastograms, in which increasing tissue hardness appears as red, green and blue in ascending order on a continuous scale [Figures 1, 2, 3, 4, 5 and 6. Therefore, the examiner receives information about the mechanical properties of the tissue.

### Statistical analysis

Microsoft® Office Excel® 2007 (Microsoft Corporation) was used for data collection. The analysis was performed with MedCalc® 7.6 statistical software (MedCalc Software bvba, Belgium). The Student's t-test was used for continuous data and comparison of means. Ultrasonographic features of benign and malignant LNs were compared using Fisher's exact test for univariate distributions. The predominant colors in the elastograms were compared using Yates' chi-square test for multivariate distributions of categorical data. When Yates' chi-square test was found to be significant, pairwise comparisons were performed using Fisher's exact test. For the calculation of 95% confidence levels we used Newcombe intervals with continuity correction [43]. Specimen histology was the gold standard for the definition of metastatic LNs. Statistical significance was assumed at p<0.05 for all tests.

## Results

We analyzed 165 healthy LNs (group 1) and 15 metastatic LNs (group 2). The breast cancer patients (group 2) were significantly older (58.3 ± 7.4 versus 50.2 ± 12.9 years, p=0.017), and had a significantly higher body mass index (28.0 ± 5.2 versus 24.8 ± 4.5 kg/m^2^, p=0.012) than the healthy probands (group 1). There was no significant difference between the groups regarding the clinical presentation of the LNs (i.e. palpable mass 13.3% versus 11.6%, p=0.690, and painful palpation 0% versus 2.4%, p=1.000).

### B-mode features and Doppler features of healthy and metastatic lymph nodes

Regarding the horizontal size of the LNs and the diameter of the medulla, there were no significant differences between the groups. Nevertheless, the vertical dimension of metastatic LNs was significantly higher (9.2mm versus 7.2mm, p=0.013). Focusing on the cortex, we found a significantly broader cortex for the metastatic LNs (4.2mm versus 1.4mm, p<0.001). Consequently, the cortex-to-medulla-ratio as well as the vertical-to-horizontal-size were significantly higher in the metastatic group (p<0.001 and p=0.002, respectively). A cortex greater than 3mm was found in only 3.1% of the healthy LNs, compared to 40.0% of the metastatic LNs (p<0.001). The results are shown in Table 1.

**Table 1:** B-mode features and Doppler sonography of healthy and metastatic LNs (mean ± standard deviation, n.s. = not significant, LN = lymph node)

**LN characteristics**	**Group 1 healthy LNs**	**Group 2 metastatic LNs**	**p**
**n**	165	15	
**Distance from the skin (mm)**	13.5 ± 5.1	15.5 ± 3.0	n.s. (0.126)
**Horizontal size (mm)**	15.8 ± 6.4	14.4 ± 7.0	n.s. (0.406)
**Vertical size (mm)**	7.2 ± 3.0	9.2 ± 3.5	0.013
**Vertical-to-horizontal-size (ratio)**	0.50 ± 0.24	0.70 ±0.26	0.002
**Cortex (mm)**	1.4 ± 0.7	4.2 ± 4.7	<0.001
**Medulla (mm)**	4.8 ± 2.4	4.1 ± 2.1	n.s. (0.299)
**Cortex-to-medulla (ratio)**	0.39 ± 0.31	1.22 ± 1.75	<0.001
**Cortex >3mm**	3.1%	40.0%	<0.001
**Architectural distortions**	0.6%	40.0%	<0.001
**Pathologic vascularization**	4.4%	14.3%	n.s. (0.109)

### Elastograms of healthy and metastatic lymph nodes

Focusing on the group of healthy LNs (n=165), the predominant color of the cortex was yellow in 1.2%, green in 13.9%, turquoise in 64.2% and blue in 20.6% of the cases respectively, and never red [Table 2]. The medulla exhibited a similar distribution of the colors (3.0%, 15.8%, 73.9% and 73.2%, respectively, never red) [Table 3]. Nevertheless, the cortex and medulla color distributions were significantly different in the multivariate analysis (p=0.004), and the pairwise comparison revealed that the cortex was significantly more often described as blue (i.e. hard) than the medulla (p<0.001).

**Table 2:** Predominant color of the cortex in sonoelastography with respect to healthy and metastatic LNs

**Cortex**	**Group 1 healthy LNs n=165**	**Group 2 metastatic LNs n=15**	**p (pairwise comparison)**
**red (soft)**	0%	0%	n.a.
**yellow**	1.2%	0%	n.s. (1.000)
**green**	13.9%	0%	n.s. (0.223)
**turquoise**	64.2%	40.0%	n.s. (0.093)
**blue (hard)**	20.6%	60.0%	0.001
**p-value (multivariate analysis)**	0.006		

The cortex of metastatic LNs is significantly harder and therefore significantly more often described as blue than the cortex of healthy LNs. (n.s. = not significant, n.a. = not applicable, LN = lymph node).

**Table 3:** Predominant color of the medulla in sonoelastography with respect to healthy and metastatic LNs

**Medulla**	**Group 1 Healthy LNs n=165**	**Group 2 Metastatic LNs n=15**	**p (pairwise comparison)**
**red (soft)**	0%	0%	n.a.
**yellow**	3.0%	6.7%	n.a.
**green**	15.8%	33.3%	n.a.
**turquoise**	73.9%	53.3%	n.a.
**blue (hard)**	7.3%	6.7%	n.a.
**p-value (multivariate analysis)**	n.s. (0.281)		

There is no difference between the two groups. (n.s. = not significant, n.a. = not applicable as the multivariate analysis was negative, LN = lymph node).

Focusing on the group of metastatic LNs (n=15), the predominant color of the cortex was either turquoise (40.0%) or blue (60.0%) but never yellow, green or red [Table 2]. The medulla was yellow in 6.7%, green in 33.3%, turquoise in 53.3% and blue in 6.7% of cases, respectively [Table 3]. Accordingly, the difference between the cortex and the medulla was statistically significant in the multivariate analysis (p=0.005).

Comparing the two groups, there was no difference regarding the color distribution of the medulla [Table 3]. However, we found a significant difference regarding the color distribution of the cortex (p=0.005). Compared to healthy LNs, the cortex of metastatic LNs was significantly more often blue (60.0% versus 20.6%, p=0.005) [Table 2].

### Sensitivity and specificity of B-mode ultrasound, Doppler ultrasound, sonoelastography and clinical examination

Analyzing the performance of single criteria, a cortex broader than 3mm in B-mode ultrasound yielded an excellent specificity (96.8%) and a low sensitivity (40.0%). Concerning sonoelastography, we applied the criterion of a blue cortex and achieved a well-balanced specificity of 79.6% and a sensitivity of 60.0%.

In order to explore the combinations of different ultrasound criteria, we combined the B-mode feature ″cortex broader than 3mm″ and the elastographic feature ″blue cortex″. In the disjunctive combination (LNs that fulfill at least one criterion were regarded as positive)**,** the specificity was 77.5% and the sensitivity was higher than with any other criterion, namely 73.3%. In the conjunctive combination (only LNs that fulfill both criteria were regarded as positive), the specificity reached an excellent level of 99.3% (higher than with any other criterion) and the sensitivity was 26.7% [Table 4].

**Table 4:** Sensitivity and specificity of conventional ultrasound, Doppler and sonoelastography for the assessment of axillary LNs including conjunctive and disjunctive combinations (95% confidence intervals in brackets)

**Prediction of LN status**	**Sensitivity**	**Specificity**
**B-Mode-US: Cortex >3mm**	40.0	96.8
	(17.5-67.1)	(92.5-98.8)
**Doppler-US: Pathologic vessels**	14.3	95.6
	(2.5-43.9)	(90.8-98.1)
**Clinical examination: Palpable LNs**	13.3	88.4
	(2.3-41.6)	(82.3-92.7)
**Elastogram: Cortex ″blue″**	60.0	79.6
	(32.9-82.5)	(72.2-85.5)
**Disjunctive combination: Cortex >3mmor″blue″ in the elastogram**	73.3	77.5
	(44.8-91.1)	(69.8-83.7)
**Conjunctive combination: Cortex >3mmand″blue″ in the elastogram**	26.7	99.3
	(8.9-55.2)	(95.8-100.0)

### Model calculation concerning the diagnostic performance of B-mode ultrasound and sonoelastography

Calculation of the negative and positive predictive values (NPV, PPV) should be based on the particular prevalence in the observed collective. The prevalence of LN metastases in individual subgroups is dependent on the tumor stage, among other factors [44-46]. In mixed collectives, the prevalence of LN metastases is estimated to be about 45% [47], which is concordant with our collective (45.5%). In particular, tumors categorized as T1 show LN metastases in about 25.9% of cases, whereas in T2 tumors, LN metastases occur in about 48.2% [48]. Based on the prevalence of LN involvement within these two risk groups, the following predictive values result:

In T1 tumors (with an estimated prevalence of LN metastases of 25.9%), the best B-mode criterion (cortex >3mm) can be expected to yield a PPV of about 81% and an NPV of ~82%. The conjunctive combination with the best elastographic criterion (blue cortex) leads to an improved PPV of ~93% with little effect on the NPV (~79%).

In T2 tumors (with an estimated prevalence of LN metastases of 48.2%), B-mode ultrasound can be expected to have a PPV of ~92% and a NPV of ~63%. The conjunctive combination with sonoelastography improves the PPV (~97%), but also impairs the NPV (~59%).

## Discussion

Sonoelastography only offers a relative measurement of tissue stiffness and is dependent on the surrounding tissue [49]. We propose a relatively simple criterion (i.e. blue cortex) as the most suitable predictor of malignancy in LNs. The fact that the cortex of metastatic LNs is significantly harder than the cortex of healthy LNs is reflected in the predominance of the colors blue and turquoise in the elastograms. Applying this single criterion, the examination with sonoelastography resulted in an SE of 60.0% and an SP of 79.6%.

However, the combination of various criteria from several imaging methods is known to improve the performance. This principle is also used in breast diagnostics, when different ultrasound features of a lesion are combined**,** or mammography and MRI are added [50]. Consequently, we combined our best B-mode criterion and the most plausible elastographic criterion in order to investigate the effect on SE and SP. The conjunctive combination of B-mode and sonoelastography resulted in an improved performance. Due to the high specificity of the method, the PPV increased, while the effect on the NPV was only marginal and without clinical relevance.

However, a false negative preoperative evaluation usually results in the resection of a metastatic involved sentinel node. This scenario implies no relevant risk to the patient. On the other hand, a false positive evaluation of axillary LN status may result in an unnecessary axillary dissection instead of sentinel node biopsy with a potentially increased morbidity. Therefore, a beneficial effect of the complementary use of sonoelastography is very likely. We propose that these aspects should be investigated further.

### Literature overview

Concerning breast masses, a scoring system (the so-called Tsukuba Elasticity Score, Itoh Score or Elasticity Score) is commonly used, which refers to the distribution of different colors within a lesion [51]. Obviously, this scoring system was developed for breast lesions and is not applicable to LNs.

For the elastographic assessment of cervical LNs, Lyshchik et al. determined an individual four-point rating scale including the visibility, relative brightness, margin regularity, and margin definition of the LNs in the elastogram. In the evaluation of 141 patients, they described an SP of 98%, an SE of 85% and an accuracy of 92% [27].

Saftiou et al. reported on cervical, mediastinal and abdominal LNs examined with endoscopic ultrasound elastography. The evaluation of the pictures was performed using a pattern analysis with RGB channel histograms. In their collective study of 42 LNs, they achieved an SP of 94.4% and an SE of 91.7% [52].

Taylor et al. performed sonoelastography in 50 breast cancer patients. They evaluated the LNs in the elastogram with either an individual visual scoring system or an individual strain scoring system. The authors described an SE and SP of 76% and 78% for conventional ultrasound, 90% and 86% for visual scoring, and 100% and 48% for strain scoring, respectively [36].

Alam et al. published data on cervical LNs in 85 patients. The authors analyzed the distribution and percentage of the LN area with high elasticity (i.e. hard, blue), with pattern 1 being an absent or very small hard area and pattern 5 indicating a hard area occupying the entire LN. The cutoff line for reactive versus metastatic was set between patterns 2 and 3. The authors reported an SE of 83% and an SP of 100% [28].

Choi et al. modified this system and classified 64 axillary LNs using a 4-point color scale based on the percentage and distribution of the LN areas with high elasticity (i.e. hard, blue). They achieved an SE of 80.7% and an SP of 66.7% [35]. These results do not fully comply with the previously described studies and our own results, as a high SP and a moderate SE is usually observed in elastography.

Generally, the performance of sonoelastography is remarkably good in studies from the literature. Nevertheless, we have to consider that these data are from dissimilar, small patient collectives, the LNs are examined in different regions of the body, and the methods show relevant variations. Therefore, more advanced comparisons of the data are not possible.

Despite these reports, we have chosen a different approach for the evaluation of the elastograms, as we propagate the idea that the cortex and the medulla of an LN should be evaluated separately. Furthermore, we tried to avoid cumbersome scoring systems. For the evaluation of the elastograms we used a simple 5-point color scale describing the predominant color of the distinct structure (red, yellow, green, turquoise, or blue) as it appears in the elastogram.

Our approach is concordant with the preliminary results of Giovannini et al., who investigated LNs with endoscopic sonoelastography in 49 patients. The authors described a high SE (100%) and a moderate SP (50%) for sonoelastography using the criterion of a homogeneously blue cortex [33].

Metastases develop preferentially in the LN cortex and cause tissue alterations. As demonstrated by our results, sonoelastography seems to be capable of detecting these minute changes in elasticity distribution, although the LN cortex only constitutes a tissue structure of a few millimeters in size.

Another option for the interpretation of elastograms is the calculation of the strain-ratio [24,25]. This mode has not been systematically analyzed in LNs and could be a matter for future research.

### Limitations of our study

The main limitation of our study is that we have no validated criteria for the description of LNs in the elastogram. Accordingly, the analysis of the predominant color is, to a certain degree, observer-dependent, as it is based on image interpretation. Nevertheless, the evaluation of B-mode images is also observer-dependent and a matter of subjective interpretation. To minimize this limitation, we chose a simplified evaluation algorithm based on five categories (predominant color described as red, yellow, green, turquoise, or blue). The analysis of inter-observer concordance could be a matter for future research.

Furthermore, the still image of the elastogram is randomly depicted by the examiner during the real-time examination. This implies the risk of an observation bias. Nevertheless, this is unavoidable and has proven stable results in previous elastographic studies of LNs [52].

Finally, the analysis of SP, PPV and NPV is limited by the fact, that a group of healthy women is probably not the optimal choice for the control group, as lymph node morphology may differ even between healthy women and node negative breast cancer patients. Furthermore, there are vast confidence intervals around parameter estimated due to the small sample size. Further studies with larger collectives consisting exclusively of breast cancer patients may yield more accurate results.

## Conclusion

– The cortex of healthy LNs is typically harder (i.e. has a higher elasticity) than the medulla.

– The cortex of malignant LNs is typically harder (i.e. has a higher elasticity) than the medulla.

– Comparing healthy LNs and metastatic LNs, the cortex of metastatic LNs is significantly harder (i.e. has a higher elasticity) than the cortex of healthy LNs.

– The definition of a blue cortex in the elastogram as a criterion for malignancy is feasible.

– Concerning the prediction of LN status, the combination of B-mode ultrasound with sonoelastography may be superior to B-mode ultrasound alone.

– The best specificity (99.3%) may be achieved by conjunctively combining B-mode ultrasound with the elastogram (cortex >3mm and cortex blue), although the sensitivity is low in this setting (26.7%).

– The conjunctive combinations of B-mode ultrasound and sonoelastography may improve the PPV (i.e. reduced false positive rate), but there may be an impairment of the NPV (i.e. increased false negative rate).

– Sonoelastography of axillary LNs must still be regarded as an experimental method. Nevertheless, in the hands of an experienced sonographer, the method of real-time sonoelastography may provide useful information about axillary LNs even today.

## Abbreviations

ALND: Axillary lymph node dissection; CT: Computer tomography; LN: Lymph node; MRI: Magnetic resonance imaging; n.a.: not applicable; n.s.: not significant; NPV: Negative predictive value; PET: Positron emission tomography; PPV: Positive predictive value; SE: Sensitivity; SNB: Sentinel node biopsy; SP: Specificity.

## Competing interests

The author's declare that they have no competing interests.

## Authors' contributions

SW contributed to the conception and design of the study and WS provided methodological advice. JD performed the ultrasound examinations and data collection. SW and JD contributed to the analysis and interpretation of the data and the writing of the manuscript. MC contributed to the writing and the reviewing of the manuscript. FD, PH and SW conducted the final review of the data and the manuscript. SW, JD, WS and MC were employees at the University Hospital of Saarland at the time of the study. All authors read and approved the final manuscript.

## Pre-publication history

The pre-publication history for this paper can be accessed here:

http://www.biomedcentral.com/1471-2342/12/35/prepub
